# Subtype of the Freezing of Gait and Motor Function in Patients with Parkinson's Disease

**DOI:** 10.1002/mdc3.70260

**Published:** 2025-07-30

**Authors:** Katsuya Sakai, Tsubasa Kawasaki, Kichol Kim, Jyunya Ogawa, Yuya Yamaguchi

**Affiliations:** ^1^ Graduate School of Human Health Sciences, Tokyo Metropolitan University Tokyo Japan; ^2^ Department of Physical Therapy School of Health Sciences, Tokyo International University Kawagoe Japan; ^3^ Department of Rehabilitation Kawaguchi Neurosurgery Rehabilitation Clinic Osaka Japan; ^4^ Department of Rehabilitation PDit Studio Tokyo Japan

**Keywords:** Parkinson's disease, subtype, freezing of gait, motor function, cluster analysis

## Abstract

**Background:**

The association between freezing of gait and motor function in Parkinson's disease is variable, and the results suggest the existence of subtypes.

**Objectives:**

This study aimed to identify the subtypes of freezing of gait and motor function in patients with Parkinson's disease.

**Methods:**

Sixty‐one patients were included. This study assessed motor function using the freezing of gait questionnaire, the Unified Parkinson's Disease Rating Scale Part III. For statistical analyses, we conducted a cluster analysis using the freezing of gait and motor function. Analysis of covariance with age and L‐dopa dosing as a covariate was performed for each cluster (*P* < 0.05).

**Results:**

Cluster 1 (*N* = 15) had the lowest freezing of gait scores compared to the other clusters, and its motor function scores were lower than those of cluster 3 (*N* = 9) (*P* < 0.05). Cluster 2 (*N* = 37) had moderate freezing of gait scores (vs. cluster 1, *P* < 0.05), and motor function showed lower scores than cluster 3 (*P* < 0.05); however, the score was not significantly different from that of cluster 1 (*P* > 0.05). Cluster 3 had moderate freezing of gait scores (vs. cluster 1, *P* < 0.05; vs. 2, *P* > 0.05) and higher motor function scores than the other clusters (*P* < 0.05); however, the freezing of gait score was not significantly different from that of cluster 2 (*P* > 0.05).

**Conclusions:**

This study revealed new subtypes that presents with freezing of gait but high motor function in patients with Parkinson's disease.

Parkinson's disease (PD) is a progressive neurodegenerative disease that causes motor and non‐motor symptoms.^1^ Motor symptoms include rigidity, tremors, balance impairment, and gait impairment, which can affect patients’ activities of daily living (ADL) and reduce their quality of life.^2^, ^3^

Freezing of gait (FOG) is one of the most common gait impairments in patients with PD.^4^ Previous studies have reported that the prevalence of FOG ranges from 50.6%.^5^ Recently, it has been suggested that there are several subtypes of FOG and that different triggers may cause them.^5^ Martens et al used data‐driven cluster analysis on 41 patients with PD. They found that there are three types of freezing: one induced by motor asymmetry, one induced by anxiety, and one induced by attention deficit.^5^ Four neurophysiological hypotheses have been proposed to explain the induction of FOG.^6^ The first is the threshold model, in which FOG is caused by progressive motor impairment. The second is the interference model, in which the simultaneous processing of motor and cognitive processes leads to overloading of the basal ganglia, causing difficulties in information processing. The third is a cognitive model, in which FOG is induced in association with executive dysfunction. The fourth is the decoupling model, in which a motor program induces FOG.^6^ It is possible that the subtypes described by Martens et al are induced by one of these models and manifest as a common symptom.

Although FOG often interferes with patients’ ADLs as a motor impairment, it has been reported to be associated with motor function in patients with PD.^7^, ^8^ Aktürk et al reported that FOG and Unified Parkinson's Disease Rating Scale (UPDRS) Part III were associated with motor function in patients with PD.^7^ They reported that FOG was weakly to moderately positively correlated with UPDRS Part III. In addition, Sakai et al also investigated the relationship between FOG and UPDRS Part III and reported a weak‐to‐moderate relationship.^9^ On while others reported that FOG was not associated with motor function in patients with PD.^10^, ^11^, ^12^ These results indicate that the degree of FOG and motor function vary, and we hypothesized that there may be several subtypes of FOG and motor function in patients with PD. The presence of subtypes in terms of the degree of FOG and motor function suggests that there are groups with high motor function even if they show severe FOG and groups with low motor function even if their FOG is mild. This differentiation in subtypes suggests the need to change the rehabilitation intervention strategies for each subtype and the possibility of providing more individualized and effective rehabilitation. Therefore, this study aimed to identify the subtypes of FOG and motor function in patients with PD.

## Methods

### Participants

This cross‐sectional study was conducted between November 2023 and July 2024. The inclusion criteria were as follows: (1) diagnosis of PD by a neurologist, (2) ability to walk with or without assistance, (3) age > 18 years, and (4) absence of lower limb orthopedic disease. The exclusion criteria were: (1) diagnosis of dementia, (2) presence of a visual field defect, and (4) diagnosis of other neurological pathologies. For the sample size of partial correlation analysis was calculated using a power of 0.95, *α* = 0.05, and a correlation coefficient of 0.56 using SPSS (version 29.0; SPSS Inc., Chicago, IL, USA).^8^, ^9^ Therefore, the sample size was more than 35 participants. Furthermore, since this study conducting cluster analysis, the sample size for cluster analysis was calculated using the formula (*N* > 2^*p*^) for the number of variables based on rules of thumb,^13^, ^14^ and since the number of variables (^*p*^) used for the cluster is 2. So, one cluster of the sample size is more than four participants. The aim of the study was explained to the participants, and written informed consent was obtained before the study was initiated. This study was approved by the Ethics Committee of Tokyo Metropolitan University (approval number: 23059) and was publicly registered in the UMIN Clinical Trials Registry (trial registration ID: UMIN000052135). The study was conducted according to the ethical standards of the 1964 Declaration of Helsinki.

### Assessments

Assessments were measured within 10 days, and participants were assessed during the “on” phase. Assessments consisted of the FOGQ, UPDRS, the Two‐step Test (TST), and the Fall Efficacy Scale (FES). In addition, L‐dopa dosing was obtained from the patient's medical records.

The Japanese version of the FOGQ is a six‐item questionnaire that assesses the degree of subjective FOG.^15^ Each item is rated on a scale of 0–4 points, with a total score of 24 points. Higher FOGQ scores indicate more severe FOG.

The Japanese version of the UPDRS consists of four parts: Part I assesses mental function, behavior, and mood; Part II assesses ADL; Part III assesses motor function; and Part IV assesses treatment complications. The total UPDRS score was 200 points, with parts I, II, III, and IV consisting of 16, 52, 108, and 24 points, respectively.^16^ Higher UPDRS scores indicated increased PD severity.

The TST is an assessment tool that reflects walking function.^17^ The participants took a maximum of two steps forward from the line of measure delineated by the assessor to measure the TST distance. The TST distance was then recorded.^17^ The TST distance was measured from toe‐to‐toe, and an assessor measured the maximum two‐step distance. The TST was performed twice.

The Japanese version of the FES assesses the respondents’ fear of falling in daily life.^18^ It consists of 10 items rated on a scale of 0 to 4 points, with a total score of 40 points. Lower FES scores indicate a higher fear of falling.

### Data and Statistical Analysis

First, we calculated the median, minimum, and maximum values of FOGQ and UPDRS Part III. Then, to investigate the correlation, we calculated the partial correlation coefficient with age as a covariate. Cluster analysis (Ward's method) was then performed using the FOGQ and UPDRS Part III. Ward's method is a type of hierarchical cluster analysis and a method for integrating data.^13^, ^19^ When integrating data, groups are formed in a way that minimizes the increase in variance (ie, variables with similar values are grouped together and brought together). A dendrogram is used to determine the clusters. The number of clusters is adopted as the appropriate number of clusters just before the nodes of the dendrogram change sharply.^13^, ^19^ This study used two variables, the FOGQ and UPDRS Part III, based on the hypothesis that previous studies has reported a low correlation between FOGQ and motor function,^8^, ^9^ and this suggests the existence of a subgroup with a low correlation between FOGQ and motor function. The reason for selecting cluster analysis is that when classifying groups, if the cutoff values and severity scores for FOG and UPDRS can be clearly calculated, classification based on those criteria is possible. However, these values have not been reported in previous studies. In such cases, cluster analysis is effective because it allows participants with similar scores to be grouped together. We used a one‐way analysis of variance to compare whether there were differences between the clusters for basic attributes. The Chi‐square test was used to determine differences in sex. One‐way analysis of covariance (ANCOVA) was used with age and L‐dopa dosing as a covariate to compare clusters for physical assessments. The Bonferroni method was used for multiple comparisons of the three clusters (*P* = 0.05/3 [0.0167]). In addition, a partial correlation analysis of the association between FOGQ and UPDRS Part III scores in each cluster was performed with age as a covariate. Statistical analysis was performed using SPSS (version 29.0; SPSS Inc., Chicago, IL, USA), and statistical significance was set at *P* < 0.05.

## Results

A total of 61 patients with PD were participated in this study (mean age: [71.5 ± 9.8]) years; sex: 35 females; Hoehn & Yahr (H&Y) stage: 3 (1–4); (Table 1).

**TABLE 1:** The characteristics of overall participants and three clusters

Variables	Overall (*N* = 61)	Cluster 1 (*N* = 15)	Cluster 2 (*N* = 37)	Cluster 3 (*N* = 9)	*F* value	*P* value
Age (year)	71.5 ± 9.8	68.7 ± 11.8	71.2 ± 9.2	77.3 ± 6.4	2.3	0.105
Male / Female	26/35	4/11	18/19	3/6	‐	0.303
BMI (k/m2)	21.6 ± 3.7	22.5 ± 0.9	21.6 ± 0.6	19.9 ± 1.2	1.3	0.262
MMSE (points)	27.9 ± 2.8	27.9 ± 3.4	28.2 ± 1.9	26.2 ± 2.0	2.6	0.089
Hoehn & Yahr	3 (1–4)	3 (1–4)	3 (1–4)	3 (2–4)	1.1	0.335
L‐dopa dose (mg/day)	444.1 ± 318.6	263.5 ± 229.1	513.5 ± 343.1	460.0 ± 236.7	3.6	0.034

*Note*: Mean ± standard deviation, Median (Min‐Max).

Abbreviations: BMI, Body Mass Index; MMSE, Mini‐Mental State Examination.

**Figure 1:**
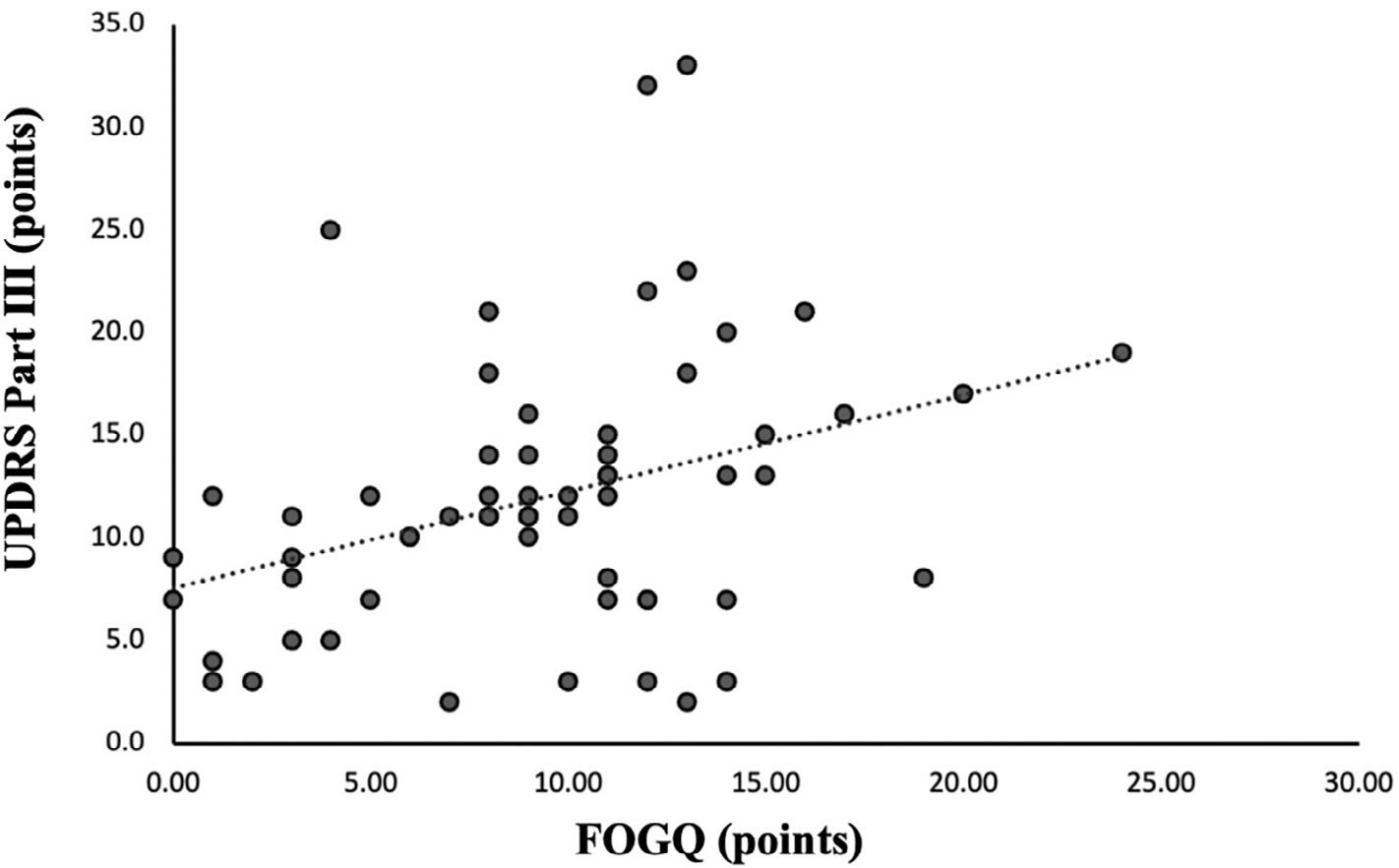
Scatter plot of FOGQ and UPDRS part III. The results of the partial correlation analysis with age as a covariate showed a significant, weak positive correlation between the FOGQ and UPDRS part III (*ρ* = 0.388, *P* = 0.003).

The FOGQ score had a median score of 10 [minimum: 0, maximum: 24], and the UPDRS Part III score had a median score of 11 [minimum: 2, maximum: 33]. The results for the partial correlation coefficient, the FOGQ, were significantly weak and positively correlated with the UPDRS Part III (*ρ* = 0.388, *P* = 0.003, Fig. 1).

As a result of cluster analysis, patients with PD were divided into three groups (cluster 1, *n* = 15; cluster 2, *n* = 37; cluster 3, *n* = 9; Table 2). Basic attributes were not significantly different among the three groups (*P* > 0.05, Table 1).

**TABLE 2:** The characteristics of overall participants and three clusters

Assessments	Overall (*N* = 61)	Cluster 1 (*N* = 15)	Cluster 2 (*N* = 37)	Cluster 3 (*N* = 9)	*P* value
FOGQ (points)	9.4 ± 5.0	3.4 ± 2.0	11.5 ± 3.8	11.7 ± 3.6	1 vs 2, *P* < 0.001 1 vs 3, *P* < 0.001 2 vs 3, *P* = 1.000
UPDRS I (points)	1.2 ± 1.6	1.1 ± 1.4	1.0 ± 1.6	2.3 ± 1.7	1 vs 2, *P* = 1.000 1 vs 3, *P* = 0.203 2 vs 3, *P* = 0.074
UPDRS II (points)	10.0 ± 5.4	7.1 ± 3.7	9.3 ± 3.9	17.3 ± 6.7	1 vs 2, *P* = 0.343 1 vs 3, *P* < 0.001 2 vs 3, *P* < 0.001
UPDRS III (points)	12.0 ± 6.7	7.7 ± 3.1	10.9 ± 4.5	23.9 ± 5.3	1 vs 2, *P* = 0.054 1 vs 3, *P* < 0.001 2 vs 3, *P* < 0.001
UPDRS IV (points)	3.4 ± 2.7	2.1 ± 2.7	3.4 ± 2.4	5.7 ± 2.9	1 vs 2, *P* = 0.190 1 vs 3, *P* = 0.001 2 vs 3, *P* = 0.014
Two‐step value	0.9 ± 0.3	1.0 ± 0.3	0.9 ± 0.3	0.6 ± 0.3	1 vs 2, *P* = 0.958 1 vs 3, *P* = 0.032 2 vs 3, *P* = 0.091
FES (points)	28.7 ± 7.2	33.4 ± 5.1	29.0 ± 6.4	19.4 ± 4.9	1 vs 2, *P* = 0.077 1 vs 3, *P* < 0.001 2 vs 3, *P* < 0.001

*Note*: Mean ± standard deviation.

Abbreviations: FES, Fall Efficacy Scale; FOGQ, Freezing of Gait Questionnaire; UPDRS, Unified Parkinson's Disease Rating Scale.

**Figure 2:**
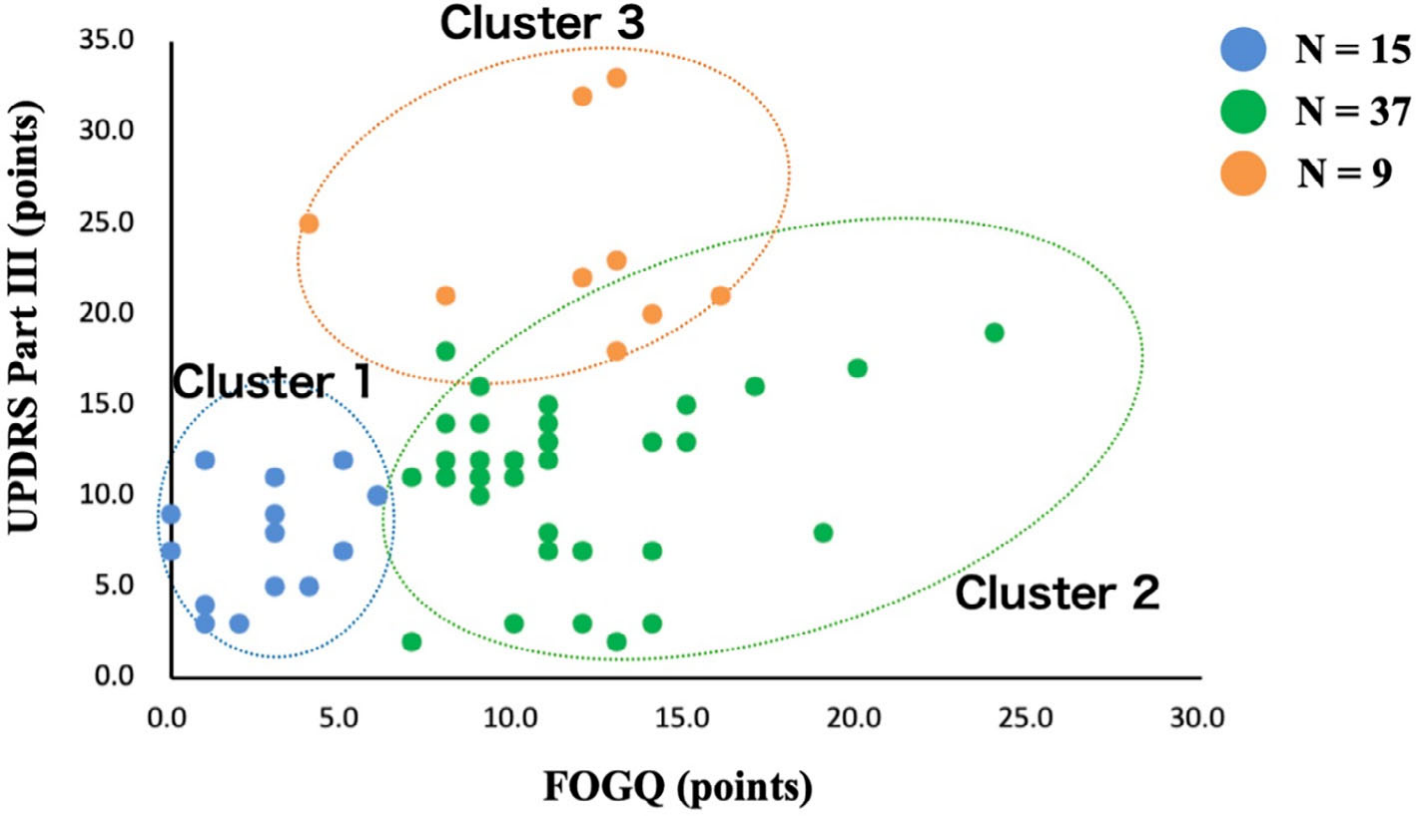
Results of cluster analysis based on FOGQ and motor function. Cluster 1 is indicated by blue dots (*N* = 15). Cluster 2 is indicated by the green dots (*N* = 37). Cluster 3 is indicated by orange dots (*N* = 9). Cluster 1 had the lowest FOGQ score and the highest motor function. Cluster 2 had moderate FOGQ and higher motor function (no difference in motor function between clusters 1 and 2). Cluster 3 exhibited moderate FOGQ and motor function.

The FOGQ score was observed main effect (*F*_2,60_ = 9.7, *P* < 0.001). The results of multiple comparisons using the Bonferroni method showed that the FOGQ score of Cluster 1 was significantly lower than those of Clusters 2 and 3 (Cluster 1 vs. Clusters 2 and 3: *P* < 0.001, Fig. 2). However, the FOGQ scores of Clusters 2 and 3 were not significantly different (*P* = 1.000). UPDRS Part II, III, IV, FES scores were observed main effect (Part II: *F*_2,60_ = 4.5, *P* < 0.001, part III: *F*_2,60_ = 13.6, *P* < 0.001, part IV: *F*_2,60_ = 2.6, *P* = 0.024, FES: *F*_2,60_ = 6.1, *P* < 0.001). The results of multiple comparisons showed that UPDRS Part II, III, IV of Cluster 1 were significantly lower than Cluster 3 (Part II, III, IV: *P* < 0.001), and Cluster 2 was significantly lower than Cluster 3 (Part II, III: *P* < 0.001; Part IV: *P* = 0.014). However, the UPDRS Parts II, III, and IV of Clusters 1 and 2 were not significantly different (Part II: *P* = 0.343; Part III: *P* = 0.054; Part IV: *P* = 0.190). The FES score of Cluster 1 was higher than that of Cluster 3 (*P* < 0.001), and Cluster 2 had a significantly higher score than Cluster 3 (*P* < 0.001). However, the FES scores of Clusters 1 and 2 were not significantly different (*P* = 0.077). In addition, no main effect was observed for UPDRS Part I score (*F*_2,60_ = 2.0, *P* = 0.071). The TST showed a significant main effect (*F*_2,60_ = 8.8, *P* < 0.001). However, multiple comparisons showed the TST of Clusters were not significantly different (Cluster 1 vs. 2, *P* = 0.958; Cluster 1 vs 3, *P* = 0.032, Cluster 2 vs. 3, *P* = 0.091).

**Figure 3:**
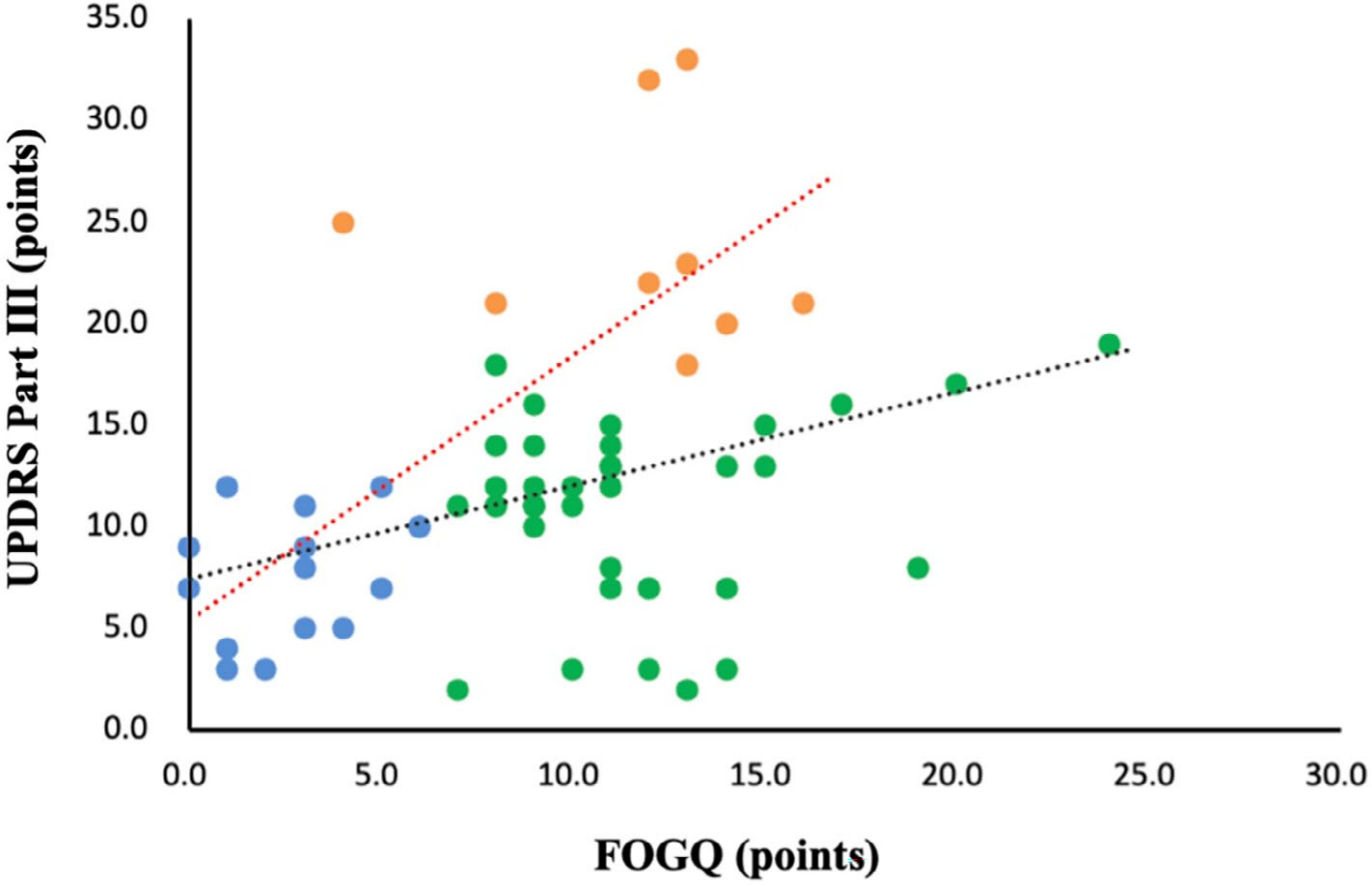
Scatter plot of FOGQ and UPDRS part III excluded Cluster 2. Cluster 1 is indicated by blue dots (*N* = 15). Cluster 2 is indicated by the green dots (*N* = 37). Cluster 3 is indicated by orange dots (*N* = 9). The black line shows the FOGQ and UPDRS Part III scores for all clusters. The red line shows the results of the partial correlation analysis with age as a covariate, excluding Cluster 2.

In addition, after excluding cluster 2, which had a similar level of motor function (UPDRS Part III) as cluster 1, we conducted age with covariate as a partial correlation analysis for the FOGQ score and UPDRS Part III score, and the correlation coefficient increased from *ρ* = 0.388 to 0.756 (*P* < 0.001, Fig. 3). The results of correlation analysis between FOGQ score and UPDRS Part III score, excluding cluster 3, showed that FOGQ score had a significant positive correlation with UPDRS Part III score (*ρ* = 0.406, *P* = 0.003). Furthermore, the results of the correlation analysis between FOGQ score and UPDRS Part III score, excluding cluster 1, showed that FOGQ score did not correlate with UPDRS Part III score (*ρ* = 0.056, *P* = 0.713).

## Discussion

This study aimed to investigate the relationship between the FOGQ and motor function and to use cluster analysis to determine whether there are subtypes of FOG and motor function in patients with PD. As a result, the correlation between the FOGQ and motor function supported previous studies,^8^, ^9^ with a weak correlation coefficient. Cluster analysis revealed the existence of three clusters. Cluster 2 was found to have moderate FOG but high motor function, where the degree of FOG did not correspond to motor function in patients with PD. When partial correlation analysis was conducted for FOGQ and UPDRS Part III, excluding this curious Cluster 2, the correlation coefficient increased (*ρ* = 0.388 to 0.756). We assumed that Cluster 2 was one of the factors that weakened or did not correlate with the correlation coefficient between FOG and motor function in patients with PD. The existence of such a curious cluster suggests that it is necessary to assess and rehabilitate patients with PD by considering both FOG and motor function.

The FOGQ showed a weak positive correlation with the UPDRS Part III. This result supported previous studies.^7^, ^9^, ^20^ The similar study by Sakai et al also investigated the association between FOGQ and UPDRS Part III,^9^ and the population and correlation values were similar to those of the present study (previous study: FOGQ score; 9.0 ± 5.0 points, UPDRS Part III; 10.6 ± 5.1 points, correlation value; *r* = 0.490, present study: FOGQ score; 9.4 ± 5.0 points, UPDRS Part III; 12.0 ± 6.7 points, correlation value; *ρ* = 0.388). Therefore, this result supports those of previous studies.^7^, ^9^, ^20^ Contrarily, some reports indicate that FOG does not correlate with motor function in patients with PD.^10^, ^11^, ^12^, ^21^ We speculated that these would indicate the presence of subtypes such as Cluster 2.

This study found that Cluster 2 had a high motor function but moderate FOG in patients with PD. In general, PD is a progressive disease, and as the motor symptoms of FOG become more severe, the associated motor function and ability to perform ADL should also decline.^2^, ^22^ However, previous studies have shown that the association between FOG and motor function is not strong.^7^, ^9^, ^20^ Furthermore, it has been reported that there is no association between FOG and motor function in patients with PD.^10^, ^11^, ^12^, ^21^ Therefore, we hypothesized that there may be subtypes, that is, groups of patients with PD whose motor function correlates with FOG and groups of patients whose motor function does not correlate with FOG. The results supported our hypothesis, and we found a cluster of individuals with moderate FOG but high motor function and ability to perform ADL in patients with PD. The correlation between FOG and motor function, excluding cluster 2, showed moderate to high correlation coefficients. This result also supports the presence of cluster 2. During the on‐phase, unlike the off‐phase, patients with PD maintain high motor function and are able to walk and perform ADLs.^23^, ^24^ However, the induction of FOG may be triggered by different factors, such as environmental factors (change of direction, starting to walk, during walking), psychological factors (anxiety, attention function), and some factors related to motor asymmetries.^5^, ^8^, ^23^ Therefore, we assumed that even if motor function was high, FOG could still be induced in patients with PD. However, the lack of group differences in UPDRS part I, which includes psychological measures, suggests that this cluster may not be psychologically induced FOG but rather environment‐dependent FOG. Therefore, we assumed that there would be a cluster of individuals with moderate FOG but high motor and ADL function among patients with PD. In addition, the high motor function of Cluster 2 was supported by the TST value.^25^ In studies measuring TST in healthy elderly participants, previous studies have reported TST values ranging from 1.54 to 1.62.^25^ In a study comparing patients with PD to older adults in need of care, prior studies reported a TST value of 1.09 for older adults in need of care, but 0.62 for patients with PD with a FOGQ near 10 points.^26^ Therefore, it is clear that Cluster 2 in this study is a group with a similar degree of motor function as the older adults who need care. Therefore, we inferred that Cluster 2 had a high motor function. Although previous studies have reported an association between FOG and fear of falling.^9^, ^27^ FOG can be performed with less fear if it is not induced in a specific location.^28^ Therefore, cluster 2 had less fear of falling than the other clusters. Another possibility is that cluster 2 was able to perform motor function tests because they retained compensatory abilities for FOG. Alternatively, they may have reported higher FOG in ADL because FOG was more pronounced. This study needed to take these possibilities into consideration. However, it should be considered that FOGQ is a subjective assessment of FOG. Goris et al reported that subjective FOG and objective FOG analysis using New‐FOGQ and video observation of rotation tasks showed discrepancies.^29^ Therefore, cluster 2, which is based on the subjective experiences of the subjects, may have weak correlations with motor function in patients with PD.

Cluster 1 and 3 were associated with the severity of FOG, motor function, ADL, and fear of falling. The correlation coefficient value excluding Cluster 2 is clearer than those including Cluster 2 (*ρ* = 0.388 to 0.756), which explains this consistency. Cluster 1 had mild FOG and better motor function. The mentioned TST values supported the high motor function.^26^ Cluster 3 has a similar FOG to Cluster 2 but lower motor function than the other clusters; the TST results show similar values to those of Sakai et al.^26^ Therefore, we assumed that Clusters 1 and 3 were associated with the FOG and motor function. In addition, Cluster 3 had the highest fear of falling, but the degree of FOG was similar to that of Cluster 2. Additionally, motor function and ADL ability were the lowest among the clusters. Therefore, fear of falling may be related to a variety of factors in addition to FOG.

### Limitations of this Study

This study has several limitations. First, we did not measure the neurophysiological findings or brain regions or neuropsychological assessments of the subtypes. Therefore, in future studies, it will be necessary to investigate the europhysiological findings or brain regions or neuropsychological assessments associated with each subtype. Second, the FOG assessment was subjective. Accelerometers or video are used for objective FOG assessment.^30^, ^31^, ^32^ Therefore, accelerometers should be considered for future studies. The third limitation is that there were few participants in Cluster 3. Future studies should include participants with more severe conditions. However, there were only a limited number of participants with severe conditions who were able to walk; therefore, Cluster 3 tended to be a small population. Fourth, the factors separating these clusters are unknown. Therefore, future studies should increase the number of participants and include a regression analysis. Finally, as mentioned in the discussion, Cluster 2 may have the ability to compensate for movement appropriately or may experience high levels of FOG in daily life. Future studies evaluating these factors may help clarify the existence of Cluster 2.

## Conclusion

This study identified an interesting cluster with moderate FOG with high motor function in patients with PD. This finding suggests that FOG and motor function in patients with PD require individualized assessment and rehabilitation.

## Author Roles

(1) Research project: A. Conception, B. Organization, C. Execution. (2) Statistical Analysis: A. Design, B. Execution, C. Review and Critique. (3) Manuscript Preparation: A. Writing of the first draft. B. Review and Critique.

K.S.: 1A, 1B, 1C, 2A, 2B, 2C, 3A,

T.K.: 1A, 1B, 1C, 2C, 3B.

K.K.: 1B, 1C, 2C, 3B.

J.O.: 1B, 1C, 2C, 3B.

Y.Y.: 1B, 1C, 2C, 3B.

## Disclosures

**Ethical Compliance Statement:** The aim of the study was explained to the participants, and written informed consent was obtained before the study was initiated. This study was approved by the Ethics Committee of Tokyo Metropolitan University (approval number: 23059) and was publicly registered in the UMIN Clinical Trials Registry (trial registration ID: UMIN000052135). The study was conducted according to the ethical standards of the 1964 Declaration of Helsinki. We confirm that we have read the Journal's position on issues involved in ethical publication and affirm that this work is consistent with those guidelines.

**Conflict of Interest and Funding sources:** No specific funding was received for this work. The authors declare that there are no conflicts of interest relevant to this work. This study was supported by the 50th Daiwa Securities Research Grant supported this research.

**Financial Disclosures for the previous 12 months:** The authors declare that there are no additional disclosures to report.

## Data Availability

The data that support the findings of this study are available from the corresponding author upon reasonable request.
